# TEACHING MOTIVATIONAL INTERVIEWING OF OLDER ADULTS IN AN INTERPROFESSIONAL EDUCATION SETTING: A PILOT STUDY

**DOI:** 10.1093/geroni/igz038.3200

**Published:** 2019-11-08

**Authors:** Nina L Tamashunas, Elizabeth Smilovich-Fine

**Affiliations:** Case Western Reserve University School of Medicine, Cleveland, Ohio, United States

## Abstract

Motivational interviewing (MI), a person-centered method of strengthening personal motivation for change, improves health behaviors (i.e. blood pressure control) in older adults. However, literature lacks exploration of teaching MI in interprofessional and geriatric settings. We sought to pilot and assess the utility of an MI curriculum for an interprofessional group of second-year graduate students (medical, physician assistant, nursing, and speech pathology) working with older adults. The 5-week curriculum included a 2-hour interactive workshop, a geriatric standardized patient (SP), and small group meetings with a geriatric community member. We employed a pretest/posttest design to assess perceived importance and confidence using a 5-point Likert scale and knowledge acquisition using a modified version of the Motivational Interviewing Knowledge and Attitudes Test. Feedback from students on curricular design was obtained via a post-curriculum survey. Of the 17 students enrolled, 100% (17/17) completed the pretest and posttest and 94% (16/17) completed the post-curriculum survey. Previous MI training ranged from 0 to 12 hours. Students felt more confident in their MI skills (p<0.05) after the curriculum. Aggregate knowledge scores did not achieve statistical significance. Feedback indicated that students enjoyed practicing with SPs and processing feedback and would have preferred more student diversity and a more advanced curriculum. While limited by sample size, this pilot demonstrated that a brief geriatric-specific curriculum was well received and improved student confidence in MI skills. Larger studies should explore tailoring teaching methods of MI knowledge and skills in a diverse learner population working with older adults to promote health behavior change.

